# Early lactate clearance is associated with biomarkers of inflammation, coagulation, apoptosis, organ dysfunction and mortality in severe sepsis and septic shock

**DOI:** 10.1186/1476-9255-7-6

**Published:** 2010-01-28

**Authors:** H Bryant Nguyen, Manisha Loomba, James J Yang, Gordon Jacobsen, Kant Shah, Ronny M Otero, Arturo Suarez, Hemal Parekh, Anja Jaehne, Emanuel P Rivers

**Affiliations:** 1 Department of Emergency Medicine, Henry Ford Hospital, Detroit, MI, USA; 2 Department of Surgery, Henry Ford Hospital, Detroit, MI, USA; 3 Department of Anesthesiology, Henry Ford Hospital, Detroit, MI, USA; 4 Department of Biostatistics and Epidemiology, Henry Ford Hospital, Detroit, MI, USA; 5 Department of Emergency Medicine, Loma Linda University, Loma Linda, CA; 6 Department of Internal Medicine, Pulmonary and Critical Care Medicine, Loma Linda University, Loma Linda, CA

## Abstract

**Background:**

Lactate clearance, a surrogate for the magnitude and duration of global tissue hypoxia, is used diagnostically, therapeutically and prognostically. This study examined the association of early lactate clearance with selected inflammatory, coagulation, apoptosis response biomarkers and organ dysfunction scores in severe sepsis and septic shock.

**Methods:**

Measurements of serum arterial lactate, biomarkers (interleukin-1 receptor antagonist, interleukin-6, interleukin-8, interleukin-10, tumor necrosis factor-alpha, intercellular adhesion molecule-1, high mobility group box-1, D-Dimer and caspase-3), and organ dysfunction scores (Acute Physiology and Chronic Health Evaluation II, Simplified Acute Physiology Score II, Multiple Organ Dysfunction Score, and Sequential Organ Failure Assessment) were obtained in conjunction with a prospective, randomized study examining early goal-directed therapy in severe sepsis and septic shock patients presenting to the emergency department (ED). Lactate clearance was defined as the percent change in lactate levels after six hours from a baseline measurement in the ED.

**Results:**

Two-hundred and twenty patients, age 65.0 +/- 17.1 years, were examined, with an overall lactate clearance of 35.5 +/- 43.1% and in-hospital mortality rate of 35.0%. Patients were divided into four quartiles of lactate clearance, -24.3 +/- 42.3, 30.1 +/- 7.5, 53.4 +/- 6.6, and 75.1 +/- 7.1%, respectively (*p *< 0.01). The mean levels of all biomarkers and organ dysfunction scores over 72 hours were significantly lower with higher lactate clearance quartiles (*p *< 0.01). There was a significant decreased in-hospital, 28-day, and 60-day mortality in the higher lactate clearance quartiles (*p *< 0.01).

**Conclusions:**

Early lactate clearance as a surrogate for the resolution of global tissue hypoxia is significantly associated with decreased levels of biomarkers, improvement in organ dysfunction and outcome in severe sepsis and septic shock.

## Introduction

The transition from sepsis to severe sepsis and septic shock is associated with a number of hemodynamic perturbations leading to global tissue hypoxia. Global tissue hypoxia accompanies a myriad of pathogenic mechanisms which contribute to the development of the multi-system organ dysfunction syndrome and increased mortality [1,2]. Although there is significant interaction between inflammation, coagulation and organ dysfunction; a clear cause and effect between global tissue hypoxia and these molecular processes leading to multi-organ failure in severe sepsis and septic shock remains unclear [3].

There is an increasing body of literature establishing the clinical utility of biomarkers as diagnostic, therapeutic and prognostic indicators in the management of patients presenting with severe sepsis and septic shock. These studies, largely derived from the intensive care unit (ICU) patient population comprise a mixed picture of pro-inflammatory, anti-inflammatory, coagulation and apoptosis biomarker responses [4,5]. However, the duration of stay for these patients prior to ICU admission whether on the general hospital ward or emergency department (ED) can be up to 24 hours [6]. Despite the abundance of knowledge in the ICU phase of severe sepsis and septic shock, little is known regarding the natural history of the biomarkers during the most proximal stage of disease presentation.

Studies targeting the early detection and eradication of global tissue hypoxia even after normalization of traditional vital signs (heart rate, blood pressure and urine output) have realized significant mortality benefit in severe sepsis and septic shock [7,8]. As a measure of tissue hypoxia and risk stratification, lactate measurements have now been incorporated into treatment protocols and care bundles [9]. We have previously reported that unresolved global tissue hypoxia reflected by inadequate lactate clearance during the early phase of resuscitation implicates organ dysfunction and increased mortality in severe sepsis and septic shock [10]. The mechanistic explanation for these observations remains un-elucidated. The purpose of this study is to examine the association of early lactate clearance with the biomarker activity of inflammation, coagulation, and apoptosis and the subsequent relationship to organ failure and outcome in early severe sepsis and septic shock.

## Materials and methods

### Study Design and Setting

This study is an analysis of biological samples prospectively collected during and after a randomized, controlled study examining early goal-directed therapy for severe sepsis and septic shock. The study was performed at Henry Ford Hospital, Detroit, Michigan, and approved by the Institution Review Board for Human Research. The details of the original early goal-directed therapy study protocol have been previously published [7].

### Patient Selection

Patients presenting to the ED of an urban academic tertiary care hospital from March 1997 to March 2001 were consented if they met enrollment criteria. Patients were included if they had 1) a source of infection suspected by the treating physician; 2) at least two of four systemic inflammatory response syndrome (SIRS) criteria [11]; and 3) either systolic blood pressure less than 90 mm Hg after a 20-30 ml/kg crystalloid fluid bolus or lactate greater than or equal to 4 mmol/L. Patients were excluded if they had age less than 18 years, pregnancy, acute cerebral vascular event, acute coronary syndrome, acute pulmonary edema, status asthmaticus, dysrhythmia as a primary diagnosis, contraindication to central venous catheterization, active gastrointestinal hemorrhage, seizure, drug overdose, burn injury, trauma, requirement for immediate surgery, uncured cancer, immunocompromised state, or do-not-resuscitate status. After meeting enrollment criteria, patients were invited to participate in the randomized protocol comparing early goal-directed therapy versus standard care and/or provide blood samples for serial biomarker measurements.

### Data Collection

Patient demographics, hemodynamic variables, laboratories, sources of infection, comorbidities, and outcome were collected at baseline. Simultaneous measurements of serum arterial lactate, biomarkers and organ dysfunction scores were obtained at time 0, 6, 12, 24, 36, 48, 60 and 72 hours after enrollment. Therapeutic interventions, such as antibiotics, fluids, packed red cells transfusion, vasoactive agents, and mechanical ventilation, given in the ED and up to 72 hours were recorded. Information required for the Acute Physiology and Chronic Health Evaluation (APACHE) II, Simplified Acute Physiology Score (SAPS) II, Multiple Organ Dysfunction Score (MODS), and Sequential Organ Failure Assessment (SOFA) score calculations were obtained at each time point. Patients were followed until in-hospital death or up to 60 days after enrollment.

### Biomarker Assays

Biomarkers were chosen to represent pro-inflammatory, anti-inflammatory, coagulation, and apoptosis pathways involved in the pathogenesis of severe sepsis and septic shock. Analysis of the biomarkers for the purpose of this study was performed from September 2003 to December 2004. The pro-inflammatory biomarkers included interleukin-6 (IL-6), interleukin-8 (IL-8), tumor necrosis factor-α (TNF-α), intercellular adhesion molecule-1 (ICAM-1), and high mobility group box-1 (HMGB-1). Anti-inflammatory biomarkers included interleukin-1 receptor antagonist (IL-1ra) and interleukin-10 (IL-10). Coagulation and apoptosis biomarkers included D-Dimer and caspase-8, respectively. Biomarker assays were performed by Biosite Inc, San Diego, California. Assays were performed using immunometric (sandwich) assays with NeutrAvidin-coated 384-well block microtiter plates (Pierce Biotechnology, Rockford, IL) and a Genesis RSP 200/8 Workstation (Tecan U.S., Durham, NC). Each sample was tested in duplicate. Before the assays, biotinylated primary antibody was diluted in assay buffer containing 10 mmol/L trishydroxymethylaminomethane HCl (pH 8.0), 150 mmol/L sodium chloride, 1 mmol/L magnesium chloride, 0.1 mmol/L zinc chloride, and 10 mL/L polyvinyl alcohol (9-10 kDa). The concentration of biotinylated antibody was predetermined by titration. The primary antibody (10 μL per well) was added to the plates and incubated. After washing, 10 g/L bovine serum albumin and 1 g/L sodium azide were added to the plate wells, which were then incubated at room temperature. Next, the plates were washed three times with borate-buffered saline containing 0.02% polyoxyethylene (20) sorbitan monolaurate (BBS-Tween).

For each sample, 10 μL aliquots were added to each plate well and the plates were incubated. Following this incubation, the plates were washed three times and alkaline phosphatase-conjugated antibody (10 μL per well) was added to each plate well and further incubated. The concentration of the alkaline phosphatase-conjugated antibody was predetermined to ensure a linear profile in the dynamic range of interest. After additional incubation, the plates were washed nine times with BBS-Tween. AttoPhos substrate (S1011, Promega, Madison, WI), a fluorescence-enhancing substrate previously diluted in AttoPhos buffer (S1021, Promega), was then added to aid in the measurement of the activity of antibody-conjugated alkaline phosphatase bound in each well. The plates were then scanned in a fluorometer (Tecan Spectrafluor, Tecan U.S.) using an excitation wavelength of 430 nm and an emission wavelength of 570 nm. Each well was scanned 6 times at 114-sec intervals, and the rate of fluorescence generation was calculated. Calibration curves were derived from eight points tested at multiple locations on the assay plate using a 4-parameter logistic fit, from which sample concentrations were subsequently calculated. Each plate included calibration wells consisting of multiple analyte concentrations and control samples. Calibration curves for each biomarker assay were generated for IL-1ra (150-30,000 pg/mL), IL-6 (20-10,000 pg/mL), IL-8 (15-3,000 pg/mL), IL-10 (15-1,000 pg/mL), TNF-α (20 -2,000 pg/mL), ICAM-1 (2.5-900 ng/mL), HMGB-1 (0.5-100 ng./mL), D-Dimer (0.5-40 μg/mL), and caspase-3 (0.1-200 ng/mL).

### Patient Stratification

*Lactate clearance *was defined as the percent change in lactate level after six hours from a baseline measurement. It is calculated by using the following formula: lactate at ED presentation (hour 0) minus lactate at hour 6, divided by lactate at ED presentation, then multiplied by 100. A positive value denotes a decrease or clearance of lactate, whereas a negative value denotes an increase in lactate after 6 hours of intervention.

The study population was sorted by increasing lactate clearance and divided into four groups with equivalent number of patients for comparisons among lactate clearance quartiles.

### Statistical Analysis

For the purpose of this study, lactate clearance, biomarkers and organ dysfunction scores were analyzed in all patients enrolled in the study, irrespective of the treatment group assigned to the patients. We a priori accepted that lactate clearance is a reflection of the therapies received by the patients, such as fluids, red cells transfusion, vasopressors, and inotrope; rather than a function of the randomization assignment to early goal-directed therapy or standard care. Descriptive statistics were used to summarize patient characteristics. The Kruskal-Wallis test was used to compare numeric variables (e.g., vital signs, hemodynamic variables, laboratories, biomarker measurements, and organ dysfunction scores over 72 hours) among patients stratified by lactate clearance quartiles. The standard Chi-square test was used to compare categorical variables (e.g., septic shock, culture status, and therapeutic interventions) among the lactate clearance quartiles. Mortality outcomes were compared among the lactate clearance quartiles using Chi-square analysis, with Kaplan-Meier estimation used to obtain mortality rates up to 12 months. A two-tailed *p-value *less than 0.05 was considered statistically significant. Data are presented as percentage or mean ± standard deviation.

## Results

Two hundred and twenty-two patients, age 65.0 ± 17.1 years, were enrolled within 1.6 ± 2.1 hours of ED presentation. The initial hemodynamic parameters included central venous pressure of 5.1 ± 8.5 mm Hg, mean arterial pressure 74.8 ± 25.7 mm Hg, central venous oxygen saturation 49.2 ± 12.6 percent, and lactate 7.4 ± 4.6 mmol/L. Fifty-five percent of patients had septic shock, 37.1% had blood culture positive, and the most common source of infection was pneumonia. Lactate clearance was 35.5 ± 43.1 percent and in-hospital mortality rate 35.0% (Table 1).

**Table 1:** Patient characteristics.

No. Patients	220
**Age (years)**	65.0 ± 17.1

**Male:Female (%)**	54.1:45.9

**Time from ED arrival to enrollment (hours)**	1.6 ± 2.1

**Length of hospital stay (days)**	13.9 ± 16.6

**Vital signs and hemodynamic variables**	

Temperature (°C)	36.3 ± 2.8

Heart rate (beats per min)	117.1 ± 30.1

Systolic blood pressure (mm Hg)	107.5 ± 36.2

Mean arterial pressure (mm Hg)	74.8 ± 25.7

Shock index (heart rate/systolic blood pressure)	1.2 ± 0.5

Respiratory rate (breaths per min)	31.5 ± 11.1

CVP (mm Hg)	5.1 ± 8.5

ScvO_2 _(%)	49.2 ± 12.6

**Laboratories**	

White blood cells (×10^3 ^per mm^3^)	14.0 ± 9.0

Hemoglobin (g/dL)	11.4 ± 2.7

Platelets (×10^3 ^per μL)	211.5 ± 122.0

Creatinine (mg/dL)	2.9 ± 2.0

Glucose (mg/dL)	259.4 ± 327.8

Anion gap (mEq/L)	21.5 ± 8.0

Total bilirubin (mg/dL)	1.5 ± 2.1

Albumin (g/dL)	2.8 ± 0.7

Lactate (mmol/L)	7.4 ± 4.6

Lactate clearance (%)	35.5 ± 43.1

**Septic shock (%)**	55.0

**Culture positive (%)**	65.6

**Blood culture positive (%)**	37.1

**Organ dysfunction scores**	

APACHE II	21.5 ± 7.0

SAPS II	49.8 ± 11.0

MODS	7.6 ± 3.1

SOFA	6.5 ± 2.9

**Source of infection (%)**	

Pneumonia	39.5

Urinary tract infection	13.2

Intra-abdominal	4.1

Other	43.2

**Comorbidities (%)**	

Chronic obstructive pulmonary disease	16.4

Chronic renal insufficiency	20.9

Congestive heart failure	30.9

Coronary artery disease	22.7

Diabetes mellitus	30.5

Hypertension	67.3

Liver disease	21.4

**Outcome (%)**	

In-hospital mortality	35.0

28-day mortality	36.4

60-day mortality	42.7

Vital signs, hemodynamic variables, laboratories and organ dysfunction scores represent baseline values at patient enrollment. ED - emergency department; CVP - central venous pressure; ScvO_2 _- central venous oxygen saturation; Acute Physiology and Chronic Health Evaluation (APACHE) II; Simplified Acute Physiology Score (SAPS) II; Multiple Organ Dysfunction Score (MODS); Sequential Organ Failure Assessment (SOFA).

The lactate clearance quartiles were -24.3 ± 42.3, 30.1 ± 7.5, 53.4 ± 6.6, and 75.1 ± 7.1%, respectively (*p *< 0.01, Table 2). There was no significant difference among the lactate clearance quartiles with respect to age, demographics, co-morbidities, blood culture positive, hemodynamic variables, baseline lactate, and other laboratories (except platelets, total bilirubin and albumin). There was significant difference in the number of septic shock patients among the lactate clearance quartiles, with the highest percent of septic shock patients in the lowest clearance quartile (*p *< 0.01). Quartiles with lower lactate clearance required significantly more vasopressor and mechanical ventilation during the first 6 hours. After 6 hours, only vasopressor remained significantly higher in lower lactate clearance quartiles (Table 3).

**Table 2:** Patient characteristics, basline vital signs, hemodynamics and laboratories by lactate clearance quartile.

	Quartile 1 N = 55	Quartile 2 N = 55	Quartile 3 N = 55	Quartile 4 N = 55	P-value
**Lactate clearance (%)**	- 24.3 ± 42.3	30.1 ± 7.5	53.4 ± 6.6	75.1 ± 7.1	<0.01

**Age (years)**	63.2 ± 16.5	68.2 ± 17.5	65.7 ± 15.7	66.7 ± 18.4	0.29

**Septic shock (%)**	70.9	58.2	54.6	36.4	<0.01

**Culture positive (%)**	45.5	33.3	42.6	32.7	0.41

**Blood culture positive (%)**	43.4	34.0	42.3	28.9	0.36

**Vital signs and hemodynamics**					

Temperature (°C)	36.3 ± 3.0	36.5 ± 3.2	36.1 ± 2.7	36.4 ± 2.5	0.67

Heart rate (beats per min)	113.8 ± 25.4	117.0 ± 27.6	120.0 ± 30.9	117.8 ± 36.0	0.70

Systolic blood pressure (mm Hg)	108.0 ± 39.7	103.3 ± 30.2	108.1 ± 35.0	110.4 ± 40.0	0.88

Mean arterial pressure (mm Hg)	76.1 ± 26.6	71.2 ± 21.8	75.8 ± 26.9	76.1 ± 27.4	0.81

Shock index (HR/SBP)	1.2 ± 0.5	1.2 ± 0.4	1.2 ± 0.5	1.2 ± 0.6	0.76

Respiratory rate (breaths per min)	29.5 ± 10.3	30.4 ± 10.9	34.2 ± 11.8	32.2 ± 10.9	0.16

CVP (mm Hg)	6.0 ± 8.7	4.5 ± 8.7	3.9 ± 8.9	5.7 ± 8.2	0.16

ScvO_2 _(%)	49.3 ± 12.9	51.4 ± 12.0	44.1 ± 12.6	51.2 ± 12.3	0.28

**Laboratories**					

White blood cells (×10^3 ^per mm^3^)	11.8 ± 7.1	13.5 ± 8.9	15.0 ± 10.5	15.6 ± 9.1	0.13

Hemoglobin (g/dL)	11.6 ± 2.6	11.0 ± 2.9	11.5 ± 2.5	11.3 ± 2.7	0.68

Platelets (×10^3 ^per μL)	163.7 ± 82.2	184.0 ± 116.7	254.1 ± 135.6	244.4 ± 124.8	<0.01

Creatinine (mg/dL)	2.5 ± 2.2	2.5 ± 1.7	2.6 ± 1.9	2.7 ± 2.4	0.94

Glucose (mg/dL)	303.5 ± 421.4	172.1 ± 150.6	240.9 ± 275.0	321.1 ± 382.0	0.07

Anion gap (mEq/L)	22.2 ± 9.6	20.0 ± 7.3	21.5 ± 7.9	22.2 ± 6.9	0.52

Total bilirubin (mg/dL)	1.9 ± 2.3	1.9 ± 3.1	1.2 ± 1.4	0.9 ± 0.7	0.03

Albumin (g/dL)	2.7 ± 0.7	2.8 ± 0.7	2.8 ± 0.7	3.1 ± 0.6	<0.01

Lactate (mmol/L)	7.5 ± 5.8	7.3 ± 4.9	7.3 ± 3.8	7.3 ± 3.5	0.47

Lactate clearance - defined as the percent change in lactate level after six hours from baseline measurement = [(Lactate^ED Presentation ^- Lactate^Hour 6^)/Lactate^ED Presentation^] × 100. A positive value denotes a decrease or clearance of lactate, whereas a negative value denotes an increase in lactate after 6 hours of intervention. Lactate clearance quartile - derived from sorting the study population by increasing lactate clearance and separating into four groups with equivalent number of patients. HR - heart rate; SBP - systolic blood pressure; CVP - central venous pressure; ScvO_2 _- central venous oxygen saturation.

**Table 3:** Therapies during the first 6 hours in the ED and from 7 to 72 hours in the ICU by lactate clearance quartile.

	Quartile 1 N = 55	Quartile 2 N = 55	Quartile 3 N = 55	Quartile 4 N = 55	P-value
**Therapies in first 6 hours**					

Antibiotics (%)	72.2	77.5	72.1	75.0	0.95

Appropriate Antibiotics (%)	86.3	77.5	90.7	88.9	0.59

Fluids (mL)	4531.2 ± 2745.3	4263.5 ± 2872.9	4266.8 ± 3449.5	3741.7 ± 3136.4	0.27

Transfusion (%)	54.6	41.8	26.4	43.6	0.27

Vasopressor (%)	50.9	36.4	27.3	14.6	<0.01

Inotrope/dobutamine (%)	5.5	3.6	9.1	12.7	0.29

Mechanical ventilation (%)	74.6	50.9	58.2	38.2	<0.01

**Therapies from 7 to 72 hours**					

Antibiotics (%)	95.0	93.9	100	97.0	0.50

Fluids (mL)	8817.2 ± 5818.1	9666.7 ± 6555.2	10329.8 ± 6866.6	7141.9 ± 4097.8	0.06

Transfusion (%)	23.6	20.0	20.0	27.3	0.77

Vasopressor (%)	47.3	47.3	30.9	18.2	<0.01

Inotrope/dobutamine (%)	10.9	14.6	9.1	12.7	0.83

Mechanical ventilation (%)	12.7	14.6	7.3	7.3	0.48

Lactate clearance quartile - derived from sorting the study population by increasing lactate clearance and separating into four groups with equivalent number of patients.

**Figure 1:**
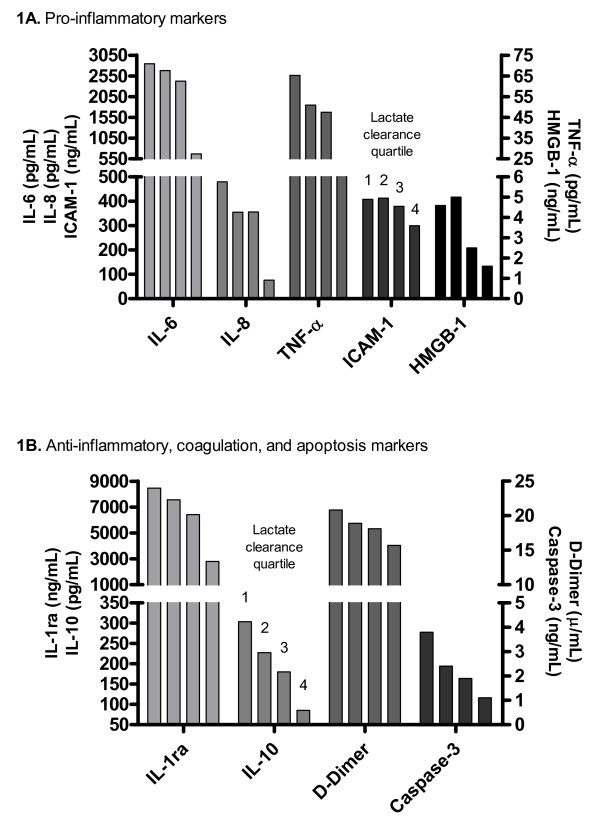
**Mean biomarker levels averaged over 72 hours based on lactate clearance quartile**. The mean levels of *pro-inflammatory markers *interleukin-6 (IL-6), interleukin-8 (IL-8), tumor necrosis factor-α (TNF-α), intercellular adhesion molecule-1 (ICAM-1), and high mobility group box-1 (HMGB-1); *anti-inflammatory markers *interleukin-1 receptor antagonist (IL-1ra) and interleukin-10 (IL-10); *coagulation marker *D-Dimer; and *apoptosis marker *Caspase-3 are significantly lower over 72 hours with higher lactate clearance quartiles.

**Figure 2:**
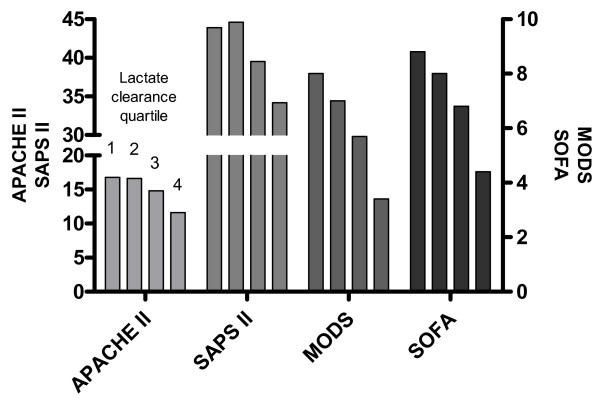
**Mean organ dysfunction scores averaged over 72 hours based on lactate clearance quartile**. The Acute Physiology and Chronic Health Evaluation (APACHE) II, Simplified Acute Physiology Score (SAPS) II, Multiple Organ Dysfunction Score (MODS), and Sequential Organ Failure Assessment (SOFA) score are significantly lower over 72 hours with higher lactate clearance quartiles.

The mean levels of all biomarkers averaged over 72 hours were significantly lower with higher lactate clearance quartiles (Table 4, Figure 1). Similarly, the mean organ dysfunction scores averaged over 72 hours were significantly lower with higher lactate clearance quartiles (Table 4, Figure 2).

**Table 4:** Biomarker levels and organ dysfunction scores averaged over 72 hours by lactate clearance quartile.

	Quartile 1 N = 55	Quartile 2 N = 55	Quartile 3 N = 55	Quartile 4 N = 55	P-value
**Biomarkers over 72 hours**					

IL-1ra (ng/mL)	8455.9 ± 8838.4	7565.4 ± 8289.1	6421.3 ± 7957.5	2792.6 ± 3635.7	<0.01

IL-6 (pg/mL)	2839.5 ± 3487.0	2680.1 ± 3174.0	2426.7 ± 3269.4	663.2 ± 1583.5	<0.01

IL-8 (pg/mL)	480.3 ± 802.4	355.3 ± 559.1	356.3 ± 735.1	76.4 ± 218.0	<0.01

IL-10 (pg/mL)	303.6 ± 298.7	227.4 ± 218.5	180.2 ± 243.4	85.4 ± 121.9	<0.01

TNF-α (pg/mL)	65.2 ± 105.9	50.9 ± 69.2	47.4 ± 72.8	19.6 ± 19.8	<0.01

ICAM-1 (ng/mL)	409.1 ± 208.1	413.3 ± 204.5	379.7 ± 213.8	299.2 ± 156.1	<0.01

HMGB-1 (ng/mL)	4.6 ± 8.3	5.0 ± 10.4	2.5 ± 3.3	1.6 ± 2.4	<0.01

D-Dimer (μ/mL)	20.8 ± 9.5	18.9 ± 9.5	18.1 ± 8.9	15.7 ± 9.7	0.04

Caspase-3 (ng/mL)	3.8 ± 7.5	2.4 ± 3.8	1.9 ± 2.6	1.1 ± 0.8	<0.01

**Organ dysfunction over 72 hours**					

APACHE II	16.8 ± 6.3	16.6 ± 6.4	14.8 ± 6.9	11.6 ± 6.0	<0.01

SAPS II	43.9 ± 12.4	44.6 ± 43.5	39.5 ± 12.0	34.2 ± 11.9	<0.01

MODS	8.0 ± 3.5	7.0 ± 4.0	5.7 ± 4.2	3.4 ± 2.5	<0.01

SOFA	8.8 ± 3.3	8.0 ± 3.4	6.8 ± 4.3	4.4 ± 2.7	<0.01

**Outcome (%)**					

In-hospital mortality	52.7	41.8	29.1	16.4	<0.01

28-day mortality	54.9	49.0	33.5	21.6	<0.01

60-day mortality	63.1	52.9	38.0	33.6	0.01

Lactate clearance quartile - derived from sorting the study population by increasing lactate clearance and separating into four groups with equivalent number of patients. IL-1ra - interleukin-1 receptor antagonist; IL-6 - interleukin-6; IL-8 - interleukin-8; IL-10 - interleukin-10; TNF-α - tumor necrosis factor-α; ICAM-1 - intercellular adhesion molecule-1; HMGB-1 - high mobility group box-1; Acute Physiology and Chronic Health Evaluation (APACHE) II; Simplified Acute Physiology Score (SAPS) II; Multiple Organ Dysfunction Score (MODS); Sequential Organ Failure Assessment (SOFA).

**Figure 3:**
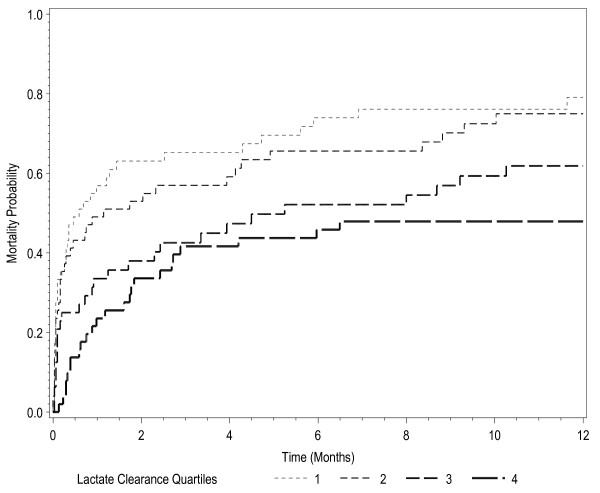
**Kaplan-Meier 12-month survival analysis based on lactate clearance quartile**. Lactate clearance quartile 1, 2, 3, and 4 have lactate clearance of -24.3 ± 42.3, 30.1 ± 7.5, 53.4 ± 6.6, and 75.1 ± 7.1%, respectively, during the first 6 hours in the emergency department (*p *< 0.01).

There was significant decreased in-hospital, 28-day and 60-day mortality for higher lactate clearance quartiles (Table 4). Kaplan-Meier survival analysis showed a survival benefit over 12 months for patients in the higher lactate clearance quartiles (Figure 3).

## Discussion

The current pathogenesis of severe sepsis and septic shock is described as a complex interaction of pro- and anti-inflammation, coagulation, and apoptosis triggered by the infecting microorganism. The bacteria outer membrane lipopolysaccharide molecule (LPS, endotoxin) activates a toll-like receptor 4 (TLR-4) signaling pathway that results in translocation of nuclear factor-κB (NF-κB) and production of inflammatory cytokines. The result is a production of pro-inflammatory cytokines that are balanced by an array of anti-inflammatory cytokines. The coagulation pathway is also activated by LPS-mediated signaling and further regulated by the cytokines, inducing the production of tissue factor, prothrombin conversion to thrombin, and fibrin production. Fibrinolysis is impaired due to increased production of plasminogen-activator inhibitor type-1 (PAI-1), decreased generation of plasmin and reduced removal of fibrin. The procoagulant state further down regulates the anticoagulant proteins, antithrombin, protein C, and tissue factor pathway inhibitor. The net result is deposition of fibrin clots throughout the endothelium, resulting in inadequate blood flow, organ hypoperfusion, global tissue hypoxia and cell death [3,12].

Clinically, lactate has been studied as a measure of illness severity in circulatory shock for several decades dating back to the 1800's [13,14]. Although there are various explanations regarding the mechanisms responsible for lactate accumulation in severe sepsis and septic shock, it remains a robust surrogate marker for the development of multi-organ failure and poor outcome [15-19]. Similar observations have been noted in other conditions of critical illness, including pediatric and adult cardiac surgery [20-22], the post-resuscitation period of cardiac arrest [23,24], trauma [25], general surgical [26], and liver surgery patients [27]. A recurring theme in these studies is the inflammatory response plays a crucial mechanistic intermediate between lactate clearance and the development of multi-organ failure.

Evidence-based guidelines have recommended that an elevated lactate is sufficient to diagnosis shock, irrespective of hypotension [28]. Sepsis with lactate level greater than or equal to 4 mmol/L is associated with high mortality and is an indication to initiate treatment protocols and care bundles [7,9,29]. We previously reported a significant inverse relationship between lactate clearance (or resolution of global tissue hypoxia) during the first 6 hours and mortality in severe sepsis and septic shock [10]. We have also shown that early goal-directed therapy targeting global tissue hypoxia to be more effective than standard care in decreasing lactate during the first six hours of intervention [7]. In this study, we found a significant association between improving lactate clearance in the first 6 hours and a corresponding decrease in mean biomarker levels over 72 hours. This potential mechanistic link was also positively associated with improved organ dysfunction scores and decreased mortality.

The association between poor lactate clearance and the need for vasopressor therapy is consistent with observations that pathogenic but reversible correlates of outcome may be established in the first few hours of disease presentation. A limited course of vasopressor therapy indicates reversible tissue hypoxia; however, prolonged vasopressor usage for hemodynamic support is associated with worse lactate clearance and thus outcome [30]. Additionally, lactate clearance has been shown to be significantly associated with improved microcirculatory flow [31]. This provides supportive evidence for the mechanistic connection between prolonged vasopressor use, tissue ischemia, persistent lactate elevation, morbidity and mortality. Our results further support the notion that tissue hypoxia plays a crucial role in the early complex mechanisms leading to the endothelial response in severe sepsis and septic shock, rather than a terminal or irreversible event following inflammation and coagulopathy [1]. Thus a goal-directed hemodynamic optimization strategy targeting the resolution of global tissue hypoxia, reflected by clearance of lactate, will likely reverse the diffuse endothelial and microcirculatory dysfunction in patients who most likely will benefit [2].

In-vitro models have shown that hypoxia induces the pro-inflammatory cytokines, IL-1, IL-6, IL-8, and TNF-α [32-36]. These cytokines then increase the expression of intercellular adhesion molecules (ICAM-1) and further activation and migration of neutrophils [37-39]. In humans, IL-6 and IL-8 elevations correlated significantly to lactate levels (as a measure of tissue hypoxia) in sepsis [40,41]. Recently, combined serial lactate and cytokine levels (IL-1, IL-6, IL-10, and HMGB-1) in septic shock patients were shown to be useful indicators of clinical outcome [42,43]. In our study, IL-1ra, IL-6, IL-8, IL-10, and TNF-α were measured due to their close association with the early pro- and anti-inflammatory response. HMGB-1 was chosen as a pro-inflammatory mediator that appears much later than the other cytokines after LPS stimulation [44]. We have shown that the higher lactate clearance in the first 6 hours, the greater the decrease in all pro-inflammatory and anti-inflammatory cytokines measured over 72 hours.

Hematologic abnormalities (leukocytotosis, anemia and thrombocytopenia) are common in severe sepsis and septic shock. Alterations in the levels of various mediators of coagulation and fibrinolysis have been reported to be associated with disseminated intravascular coagulation (DIC) and mortality [45]. Patients with SIRS and sepsis having DIC were shown to have higher serial lactate levels over 4 days compared to those patients without DIC, suggesting a pathogenic link between tissue hypoxia and intravascular coagulation [46]. While no single marker measured at hospital admission is sufficiently sensitive or specific in diagnosing DIC, we chose to measure D-Dimer as a marker of coagulation in this study as it is widely available, a correlate to the pro-inflammatory cytokine levels, and a valuable screening marker for organ failure and mortality [47-49]. It also has been used previously as an indicator of response to therapies such as recombinant human activated protein C in severe sepsis [50]. In our study, we showed that improvements in coagulation (reflected by a decrease in D-Dimer levels over 72 hours) corresponded with lactate clearance during the first 6 hours. Our results provide further evidence that tissue hypoxia may be a preceding or parallel event to the pro-coagulant state in severe sepsis and septic shock, and therapies targeting tissue hypoxia may play a crucial role in reversing this coagulopathy.

Cell death through apoptosis is a highly regulated process in the presence or absence of inflammation [51]. Apoptosis is initiated by two pathways: 1) a receptor activated, caspase-8 mediated (extrinsic) pathway; and 2) a mitochondrial initiated caspase-9 mediated (intrinsic) pathway. Either of these caspases can activate caspase-3 in the common pathway resulting in final cell death. Caspases are pro-apoptotic proenzymes that inactivate protective proteins and contribute to cell death by direct cellular disassembly via cell shrinkage (pyknosis) and nuclear fragmentation (karyorrhexis) [52]. The regulation of apoptosis in sepsis is complex, as the infecting pathogen may inhibit or induce apoptosis, involving both the extrinsic and intrinsic pathways, to enhance its damaging effects to the host [53]. Caspase activation in apoptosis is an energy-dependent process. Hypoxia can induce apoptosis as long as cells have an adequate amount of adenosine triphosphate. Previously, apoptosis was believed to occur via the intrinsic pathway with cytochrome c release and caspase-9 activation in oxygen-deprived cells [54]. However, the extrinsic pathway may also play an important role in oxidative stress induced apoptosis [53]. In this study, caspase-3 as a marker of the final common pathway in apoptosis was shown to be elevated over 72 hours in patients with decreased lactate clearance, compared to lower caspase-3 in patients with higher lactate clearance. This finding supports the premise that tissue hypoxia in severe sepsis and septic shock is associated with increased apoptosis, suggesting that the ill effects resulting in cell death may be mitigated by resolution of global tissue hypoxia.

Our results provide evidence that the design and interpretation of future clinical trials should consider the early stages of severe sepsis and septic shock. Previously, two studies failed to show significant outcome benefit with inhibition of TNF-α and IL-1ra in severe sepsis and septic shock patients enrolled in the ICU [55,56]. The association of lactate clearance with these targeted biomarkers shown in our study suggests that the severity of tissue hypoxia should be part of patient selection criteria in studies examining novel therapies that may alter its down stream effects. The failure to consider the magnitude, duration of tissue hypoxia and the timing of patient enrollment in clinical trials will likely result in some degree of hemodynamic heterogeneity confounding any treatment effect [57].

The results of our study do not confirm a causal relationship, but an association between lactate clearance in the first 6 hours and biomarker response over 72 hours. High lactate clearance quartiles had fewer patients in septic shock obviously requiring less vasopressor usage, but no difference in antibiotic and fluid administration. Lactate clearance over 6 hours may also depend on the patient's underlying comorbidities, such as liver disease, and the disease process rather than solely on the therapies themselves. However, baseline demographics, comorbidities, lactate and hemodynamic variables were similar in all quartiles. Thus the ability to clear lactate irrespective of the mechanism and its association with improved biomarkers suggests that further studies are needed to examine global tissue hypoxia as an inciting factor in the pathogenic pathways of severe sepsis and septic shock. Which of the three pathogenic pathways predominate as an association to tissue hypoxia cannot be discerned by this exploratory study. Nonetheless, our observation of a significant correlation of lactate clearance and decrease mortality is consistent with previous studies.

We have previously shown that early goal-directed therapy is significantly more effective than standard therapy in decreasing lactate (by 44% compared to 29%, p < 0.01) during the first 6 hours, resulting in improved organ dysfunction and mortality [7]. We further showed that global tissue hypoxia and early goal-directed therapy were associated with distinct biomarker patterns that were evident as early as 3 hours after intervention [2]. The purpose of this study was to show that lactate clearance is associated with improved biomarkers and organ dysfunction scores. We a priori chose not to distinguish lactate clearance, biomarker responses, and organ dysfunction scores by resuscitation groups. While we have shown that lactate clearance is a mechanistic link in the early pathogenesis of sepsis, these findings do not support the substitution of lactate clearance as an independent alternative to an organized hemodynamic optimization strategy such as early goal-directed therapy.

## Conclusions

This study showed a significant association between lactate clearance and biomarkers of pro- and anti-inflammation, coagulation, apoptosis; and further with multi-organ dysfunction and mortality in severe sepsis and septic shock. These findings support a growing body of evidence suggesting that global tissue hypoxia plays a crucial role in the complex mechanisms leading to the endothelial response in severe sepsis and septic shock rather than a terminal event. Future studies examining the pathogenic mechanisms or novel therapies for severe sepsis and septic shock should include lactate clearance as a measure of prognosis and therapeutic responses.

## Competing interests

The authors declare that they have no competing interests.

## Authors' contributions

HBN, EPR were responsible for the study design and interpretation of the data. JJY, GJ performed the statistical analyses. HBN, EPR approved the final submission of the manuscript. All authors contributed to the data collection, drafting of the manuscript and provided critical revision of the manuscript for intellectual content.
